# Effect of Structured Yoga Program on Stress and Well-being Among Frontline Healthcare Workers During COVID-19 Pandemic

**DOI:** 10.7759/cureus.43081

**Published:** 2023-08-07

**Authors:** Puneet Misra, Suprakash Mandal, Gautam Sharma, Shashi Kant, Sanjay Rai, Kapil Yadav, Meenu Sangral, Priyanka Kardam

**Affiliations:** 1 Centre for Community Medicine, All India Institute of Medical Sciences, New Delhi, IND; 2 Department of Cardiology, All India Institute of Medical Sciences, New Delhi, IND; 3 Centre for Community Medcine, All India Institute of Medical Sciences, New Delhi, IND

**Keywords:** covid-19, frontline, health-worker, stress, yoga

## Abstract

Context: Frontline healthcare workers are at risk of developing psychological distress during a pandemic. Yoga, a form of mind-body medicine can reduce body stress and increases well-being.

Aims: To assess the effect of yoga on the stress and well-being of healthcare workers during the COVID-19 pandemic.

Settings and design: This single-arm pre-post study was conducted among frontline health workers (support staff, paramedics, and medics) posted at a secondary care hospital in a North Indian district.

Methods and materials: Basic demographic details, blood pressure, anthropometric variables like height, weight, and biochemical variables like glycosylated hemoglobin (Hb1Ac), fasting and post-prandial blood sugar, lipid profile, serum cortisol, and C-reactive protein were measured. Stress levels were assessed using the depression anxiety and stress scale (DASS)-21 while well-being was assessed using the World Health Organization (WHO)-5 well-being scale. Twelve weeks of supervised yoga session was provided for 1 hour per session, 3 times per week.

Statistical analysis: The mean value was compared from baseline to post-intervention with paired t-test/Wilcoxon signed rank test.

Result: A total of 89 participants were enrolled, 53 (59.5%) being male. Two-thirds of the participants were aged 20-39 years. During follow-up, 80 participants completed 12 weeks of yoga sessions. Post-intervention DASS-21 score decreased and WHO-5 increased significantly. The glycosylated hemoglobin (HbA1c) level and cholesterol-HDL ratio decreased significantly. Other variables didn’t change significantly. No adverse effects were reported by the participants.

Conclusion: Supervised structured yoga sessions helped decrease stress, depression, and anxiety and improved well-being. Therefore, it can be a feasible strategy to manage workplace-related stress and phycological morbidities.

## Introduction

The COVID-19 pandemic impacted population health in several aspects including mental health [1]. During the rise of COVID-19 cases, anxiety, stress, and the extreme fear of catching the novel virus were faced by every section of the population, especially the frontline healthcare worker who had the highest potential for exposure. Despite preventive measures being adopted, the numbers of infected healthcare workers and deaths occurring among them around the world were alarming [2]. Healthcare workers who were directly involved in the diagnosis, treatment, and care of COVID-19 patients or other patients during the COVID-19 outbreak were at risk of developing psychological distress and other mental health symptoms [3,4]. Healthcare workers are exposed to hazards that put them at risk of infection [5]. The increasing number of confirmed and suspected COVID-19 cases, overwhelming workload, depletion of personal protection equipment, widespread media coverage, lack of specific drugs, and feelings of having been inadequately supported may have all contributed to the mental burden of these healthcare workers [4].

Cortisol is often called a "stress hormone." It is a glucocorticoid hormone that is released when the hypothalamic-pituitary-adrenal (HPA) axis is activated. Circulating cortisol levels increase in response to physical and psychological stress. It is therefore used as a biomarker of a stress response [6]. Scientists have known for years that elevated cortisol levels interfere with learning and memory, lower immune function and bone density, and increased weight gain, blood pressure, cholesterol, and heart diseases. Elevated cortisol levels act as a potential trigger for mental illness and decreased resilience [7].

Chronic stress is associated with inflammation and has been linked to increased morbidity and mortality in the aging population [8]. C-reactive protein (CRP) is considered a marker of chronic, low-grade inflammation [9,10]. Longitudinal studies have demonstrated that among generally healthy adults, high baseline CRP independently predicted the future onset of cardiovascular events and cardiac morbidity [11]. Elevated CRP concentrations have been associated with a growing number of mental conditions, including depressive syndromes and psychological distress [12-14].

Interventions aimed at reducing chronic inflammation can help attenuate chronic conditions and improve patients’ well-being [8]. Emerging evidence suggests that mind-body interventions (i.e., mindfulness-based stress reduction [MBSR], meditation, tai chi, qigong, yoga, and relaxation) have the potential to reduce circulating pro-inflammatory cytokines, but results have been mixed [8]. Complementary and alternative treatments can lower inflammation and reduce the complications of chronic illnesses and increase the quality of life [15]. Yoga is one of the most commonly used mind-body interventions. It is an ancient, Eastern tradition that usually includes regulated breathing, moving through various postures, and meditation [15].

Yoga is recognized as a form of mind-body medicine that decreases body oxidative stress and increases hypothalamic-pituitary-adrenal (HPA) axis stability [16]. These physical changes activate the parasympathetic nervous system, triggering relaxation, improving self-compassion, and decreasing stress and anxiety [17]. Reduced input of norepinephrine to the paraventricular nucleus of the hypothalamus may explain the decreased corticotrophin-releasing hormone and cortisol [18].

A previous study has suggested that the practice of yoga reduces perceived stress and negative feelings, and long-term yoga training improves stress-related psychological symptoms such as anxiety and anger [19]. Few studies report moderate improvements in health-related quality of life, fewer depressive symptoms, less trait anxiety, and greater self-efficacy after 3 months of practice [20]. There is strong evidence, derived from systematic reviews, about the positive effects of yoga practice on stress management and prevention of burnout among healthcare workers [21-23] indicating that it can be incorporated into workplace health promotion in healthcare settings [24].

Systematic reviews and yoga trials are increasing, indicating the potential increase in the quality of evidence with emphasis on stress-related disorders among the general population as well as healthcare workers. However, little is known about the level of stress on frontline healthcare workers who were directly involved in providing care to patients during the COVID-19 outbreak. Some studies have focused to evaluate the mental health outcomes among frontline healthcare workers internationally. However, to our knowledge, in India, no such study was available that assessed the effect of yoga on stress levels among frontline healthcare workers during the COVID-19 outbreak.

We, therefore, aimed to assess the effect of structured supervised yoga on stress and well-being levels among the frontline healthcare workers at a secondary care hospital.

## Materials and methods

Study design and setting

This was a single-arm pre-post interventional study, conducted among the frontline healthcare workers (support staff, paramedics, and medics) posted at a secondary care level sub-district hospital in Faridabad district of Haryana state in India. This was a 50-bedded sub-district hospital. Approval for the study was obtained from the Institutional Ethics Committee of All India Institute of Medical Sciences, New Delhi (IEC-463/22.05.2020). 

During the study period, there was regular testing for severe acute respiratory syndrome coronavirus 2 (SARS-CoV-2) infection among outdoor as well as indoor patients and for the operation theatre (OT). Though there was no inpatient admission of known COVID-19 patients, a significant number of positive SARS-CoV-2 infection were detected; thus random exposure from those patients and colleagues were a regular scenario in the hospital.

Study population

The study population was the staff of the hospital involved in patient care. The total number of staff was approximately 200 consisting of junior doctors, nursing officers, support staff, security personnel, laboratory technicians, and pharmacists. All the staff of the hospital working for at least 1 year and all those who were willing to do yoga for 3 months were included. Those who were not willing to consent to blood investigations, already diagnosed with a severe illness like cardiac disease, severe pulmonary disease, or disease of the spine where this yoga module is contraindicated were excluded.

Intervention

Regular structured yoga sessions were provided at the hospital campus at various times. Thrice a week, 1-hour-long sessions were provided at the hospital campus by a trained yoga teacher for 12 weeks. Those who completed 12 weeks of follow-up were included in the post-intervention assessment and a minimum of 24 yoga sessions attended were considered for inclusion in the post-intervention analysis. The structured yoga module was developed by trained personnel from the Centre for Integrated Medicine and Research, All India Institute of Medical Sciences, New Delhi. During the 12 weeks of the study period, the hospital's strength did not change in terms of manpower and workload.

Study variable

Basic demographic details, work-related details, height, weight, waist circumference, and clinical variables like blood pressure, heart rate, and respiratory rate were collected and the rate-pressure product was derived by multiplication of systolic blood pressure and heart rate. Levels of perceived stress, anxiety, and depression were assessed by a 21-item psychometric depression anxiety and stress scale (DASS)-21. Whereas the perceived well-being was assessed by a 5-item World Health Organization (WHO)-5 scale. Objective variables for stress biomarkers were the level of serum cortisol and high-sensitive C-reactive protein (HS-CRP). Apart from these, glycosylated hemoglobin (Hb1Ac), fasting blood glucose, postprandial blood glucose, and lipid profile were assessed. The assessment was conducted by trained field workers and biochemical tests were done by lab technicians at baseline and after the completion of the intervention.

Blood collection, transport, and analysis

Venous blood samples of 5 ml were collected from each participant in their fasting state by aseptic venipuncture of the antecubital vein and transferred to a vacutainer vial containing an anticoagulant for whole blood parameters. The sample for HbA1c, fasting blood sugar (FBS), and postprandial blood sugar (PPBS) was analyzed at the same hospital laboratory, while serum cortisol and CRP analysis were done at the cardiac biochemistry lab of the linked main campus in New Delhi.

Data analysis

All the data were entered into Excel format and were analyzed using STATA version 12 (StataCorp LP, College Station, TX, USA) software. The variables were expressed by proportion and mean scores. The outcome variables were analyzed by paired t-test to find significant changes at follow-up compared to baseline.

## Results

Out of 182 staff approached, 89 participants gave consent to participate in the study out of which 53 (59.5%) were male and 36 (40.5%) female (Table 1).

Two-thirds of the participants were within the age group of 20-39 years. The mean (SD) age of the participants was 37.1 (8.6) years. At the end of the 12 weeks of follow-up, 80 participants completed the follow-up assessment.

**Table 1:** Baseline description of basic demographic variables of all the enrolled participants

Variables		Total (n=89)
Age in years (Mean ± SD)		37.1 ± 8.6
Sex n (%)	Male	53 (59.5)
	Female	36 (40.5)
Age group n (%)	20-39	59 (66.3)
	40-59	30 (33.7)

Compared to the baseline anthropometric and clinical variables, there was a significant reduction in body weight (p=0.0001) and the rate pressure product (p=0.021). All the other variables though reduced but not significantly (Table 2).

**Table 2:** Baseline and follow-up comparison of anthropometric and clinical variables of 80 participants remaining in the study *p-value was calculated by paired t-test (80 participants of baseline vs 80 participants of follow-up) SBP: systolic blood pressure DBP: diastolic blood pressure

Variables	Baseline (n=80)		Follow-up (n=80)		p-value*
	Mean ± SD	95% CI	Mean ± SD	95% CI	
Height (cm)	164.2 ± 9.1	162.3 - 166.1	163.70 ± 9.1	161.7 - 165.7	0.889
Weight (kg)	70.3 ± 12.5	67.7 - 72.9	69.2 ± 11.9	66.5 - 71.8	0.0001
SBP (mmHg)	116.6 ± 14.1	113.6 - 119.5	115.8 ± 11.8	113.2 - 118.4	0.264
DBP (mmHg)	77.5 ± 10.0	75.4 - 79.6	77.0 ± 9.8	74.8 - 79.2	0.268
Heart rate (HR; beats/minute)	80.5 ± 11.5	78.2 - 82.9	77.8 ± 10.1	75.6 - 80.1	0.051
Rate pressure product (SBP × HR)	9387.57 ± 1907.5	8963.1 - 9812.1	9034.8 ± 1635.4	8670.9 - 9398.8	0.021

Among the biochemical variables, there was a significant reduction in HbA1c (p=0.0291) and the cholesterol-HDL ratio (p=0.043). The other variables didn’t change significantly (Table 3).

**Table 3:** Baseline and follow-up comparison of glycaemic profile and lipid profile of 80 participants remaining in the study FBS: fasting blood sugar HbA1c: glycosylated hemoglobin HDL: high-density lipoprotein LDL: low-density lipoprotein VLDL: very low-density lipoprotein Chol: cholesterol ESR: erythrocyte sedimentation rate HRP: horseradish peroxidase

Variables	Baseline (n=80)		Follow-up (n=80)		p-value
	Mean ± SD	95% CI	Mean ± SD	95% CI	
FBS (mg/dl)	92.8 ± 19.4	88.8 - 96.9	93.4 ± 19.8	88.9 - 97.8	0.575
HbA1c (%)	5.6 ± 0.9	5.4 - 5.8	5.5 ± 0.8	5.3 - 5.7	0.0291
HDL (mg/dl)	43.5 ± 9.6	41.5 - 45.5	45.4 ± 10.9	42.9 - 47.8	0.0562
LDL (mg/dl)	94.2 ± 25.2	88.9 - 99.5	93.0 ± 24.4	87.6 - 98.4	0.289
Total cholesterol (mg/dl)	166.9 ± 31.9	160.2 - 173.7	166.9 ± 30.1	160.3 - 173.6	0.574
VLDL (mg/dl)	35.7 ± 57.5	23.6 – 47.9	25.2 ± 13.7	22.1 - 28.2	0.1161
Chol: HDL	4.0 ± 1.2	3.8 - 4.3	3.9 ± 1.1	3.6 - 4.1	0.043
LDL: HDL	11.9 ± 64.4	1.5 - 25.6	2.1 ± 0.9	1.8 - 2.2	0.152
Cortisol (mcg/dl)	9.7 ± 3.9	8.8 - 10.5	10.3 ± 0.4	9.4 - 11.2	0.176
ESR (mm)	21.9 ± 10.9	19.6 - 24.4	24.2 ± 1.4	21.3 - 27.1	0.103
HRP (mg/dl)	2.9 ± 3.7	2.9 - 3.7	3.0 ± 0.7	1.6 - 4.4	0.936

There was a statistically significant decrease in the score of DASS-21 in all three domains: depression, anxiety, and stress. There was an increase in the WHO-5 score after the intervention (p<0.001) (Table 4). No adverse effects of the intervention were reported.

**Table 4:** Baseline and follow-up comparison score of DASS-21 and WHO-5 scale of 80 participants remaining in the study DASS-21: depression anxiety stress scale-21 WHO-5: 5-item World Health Organization well-being scale

Variable	Baseline (n=80)		Follow-up (n=80)		p-value
	Mean ± SD	95% CI	Mean ± SD	95% CI	
DASS-21: Depression	1.1 ± 2.7	0.5 - 1.6	0.1 ± 0.3	0.02 - 0.2	0.0017
DASS-21: Anxiety	0.8 ± 1.7	0.5 - 1.2	0.2 ± 0.7	0.05 - 0.4	0.0007
DASS-21: Stress	1.3 ± 2.5	0.8 - 1.8	0.2 ± 0.63	0.1 - 0.4	0.0002
WHO-5 score	18.9 ± 4.7	17.7 - 19.8	21.3 ± 3.1	20.6 - 22.0	<0.0001

## Discussion

Our study provided evidence that structured yoga of 12 weeks duration was associated with a reduction of both subjective as well as few objective variables in this study. The clinical variables denoting stress, like rate pressure product, changed significantly. However, there was no significant change in serum stress biomarkers. The subjective score of the DASS-21 and WHO changed significantly. During the entire 12 weeks of the follow-up period, there was no change related to the workload or number of manpower in the participants’ respective workplaces. Therefore, the improvement in overall stress, anxiety, depression, and well-being component may be attributed to the yoga intervention assuming there was no change in stressors in personal life.

Since the COVID-19 outbreak occurred, there was continuous fear among frontline healthcare workers, which had affected their mental and physical health leading to different consequences on their quality of life, and on the healthcare system itself [25]. Addressing the mental health issues of frontline healthcare workers is important for the better prevention and control of any emergency like such a pandemic. Therefore we considered this evidence as a positive effect of yoga on stress levels in frontline healthcare workers during the pandemic and it has proven to be an effective intervention in reducing stress, and anxiety and improving well-being. According to one literature review, the role of yoga during the pandemic situation has been found to have benefits in several aspects: improvement of immunity, psychological well-being, better control of pre-existing lifestyle-related diseases, and better-coping ability with stressful situations leading to improved work-life balance [26-28].

Several interventional studies done among health workers had shown the effect of yoga on stress, anxiety, depression, and well-being [21,24,29]. A study, similar to this pre-post study done in the context of the COVID-19 pandemic among 50 health workers also found significant improvement in burnout and psychological well-being [30]. Apart from this, one cross-sectional study done among 860 Brazilian health practitioners found a lower level of stress, anxiety, and depression among those practicing yoga than those who do not [31].

We did not find any effect of structured yoga on the serum cortisol, CRP, or erythrocyte sedimentation rate (ESR) level. This could be because the mean cortisol level of the participants was within the physiological limit or the non-specific nature of the markers. Yoga practice affects the stabilization of the HPA axis which normalizes the serum cortisol towards physiological limit rather than just reducing it [32]. Hence, the serum cortisol level was seen to be maintained within normal physiological limits.

Body weight decreased significantly after the intervention. Yoga has been reported to reduce body weight [33]. Though this decrease could also be due to other factors like a change of diet or physical activity. The HbA1c and cholesterol-HDL ratios decreased significantly. There is established evidence of yoga practice having a beneficial effect on HbA1c [34] and lipid profile [35]. Thus, our findings are consistent with the findings of previous studies. However, a controlled randomized study would be able to provide more robust evidence

Our study attempted to explore a possible effective intervention to improve the psychological health as well as physical health of the healthcare workers involved in hospital work during the pandemic situation. The availability of a professional yoga therapist ensured to maintenance and delivery of the standard quality of yoga sessions to all the participants throughout the study. There was a relatively lower loss to follow-up in this study compared to the usual pattern of loss to follow-up in yoga-related studies (9 were lost to follow-up out of 89 enrolled).

This study was a single-arm pre-post-design trial. We could not assess the sustainability of the practice as well as the effect after the study period. Moreover, the changing pandemic situation thus the dynamic psycho-occupational environment might imply the reproducibility of the evidence.

## Conclusions

The supervised structured yoga session helped to decrease the overall stress, depression and anxiety score as well as to increase the quality of life among frontline health workers during a pandemic situation. Though further studies can strengthen the evidence, it may be a feasible strategy to improve overall health.

## References

[1] Miyah Y, Benjelloun M, Lairini S, Lahrichi A (2022). COVID-19 impact on public health, environment, human psychology, global socioeconomy, and education. ScientificWorldJournal.

[2] Nguyen LH, Drew DA, Graham MS (2020). Risk of COVID-19 among front-line health-care workers and the general community: a prospective cohort study. Lancet Public Health.

[3] Wilson W, Raj JP, Rao S, Ghiya M, Nedungalaparambil NM, Mundra H, Mathew R (2020). Prevalence and predictors of stress, anxiety, and depression among healthcare workers managing COVID-19 pandemic in India: a nationwide observational study. Indian J Psychol Med.

[4] Lai J, Ma S, Wang Y (2020). Factors associated with mental health outcomes among health care workers exposed to coronavirus disease 2019. JAMA Netw Open.

[5] (2020). Coronavirus disease (COVID-19) outbreak: rights,roles and responsibilities of health workers, including key considerations for occupational safety and health. https://www.who.int/publications-detail/coronavirus-disease-(covid-19)-outbreak-rights-roles-and-responsibilities-of-health-workers-including-key-considerations-for-occupational-safety-and-health.

[6] Snopkowski K, Demps K, Scaggs S (201919). Small group learning is associated with reduced salivary cortisol and testosterone in undergraduate students. J. Scholarsh Teach Learn.

[7] Potey GG, Rahul V, Chanda R, Sanjeev R, Mahapatra SP (2016). Effect of yoga practices on examination stress induced changes in serum cortisol level & cardiovascular parameters in young healthy medical students.. World J Pharm Pharm Sci.

[8] Djalilova DM, Schulz PS, Berger AM, Case AJ, Kupzyk KA, Ross AC (2019). Impact of yoga on inflammatory biomarkers: a systematic review. Biol Res Nurs.

[9] Ferri C, Croce G, Cofini V (2007). C-reactive protein: interaction with the vascular endothelium and possible role in human atherosclerosis. Curr Pharm Des.

[10] Lavie CJ, Milani RV, Verma A, O'Keefe JH (2009). C-reactive protein and cardiovascular diseases--is it ready for primetime?. Am J Med Sci.

[11] Rietzschel E, De Buyzere M (2012). High-sensitive C-reactive protein: universal prognostic and causative biomarker in heart disease?. Biomark Med.

[12] Pikhart H, Hubacek JA, Kubinova R (2009). Depressive symptoms and levels of C-reactive protein: a population-based study. Soc Psychiatry Psychiatr Epidemiol.

[13] Howren MB, Lamkin DM, Suls J (2009). Associations of depression with C-reactive protein, IL-1, and IL-6: a meta-analysis. Psychosom Med.

[14] Wium-Andersen MK, Ørsted DD, Nielsen SF, Nordestgaard BG (2013). Elevated C-reactive protein levels, psychological distress, and depression in 73, 131 individuals. JAMA Psychiatry.

[15] Mongiovi J, Shi Z, Greenlee H (2016). Complementary and alternative medicine use and absenteeism among individuals with chronic disease. BMC Complement Altern Med.

[16] Chuang LH, Soares MO, Tilbrook H (2012). A pragmatic multicentered randomized controlled trial of yoga for chronic low back pain: economic evaluation. Spine (Phila Pa 1976).

[17] Aswathy S, Unnikrishnan AG, Kalra S (2013). Effective management of type 2 DM in India: looking at low-cost adjunctive therapy. Indian J Endocrinol Metab.

[18] Kumar K, Singh V, Kumar D (2018). Effect of yoga and meditation on serum cortisol level in first-year medical students. Int J Res Med Sci.

[19] Yoshihara K, Hiramoto T, Oka T, Kubo C, Sudo N (2014). Effect of 12 weeks of yoga training on the somatization, psychological symptoms, and stress-related biomarkers of healthy women. Biopsychosoc Med.

[20] Lee SW, Mancuso CA, Charlson ME (2004). Prospective study of new participants in a community-based mind-body training program. J Gen Intern Med.

[21] Cocchiara RA, Peruzzo M, Mannocci A (2019). The use of yoga to manage stress and burnout in healthcare workers: a systematic review. J Clin Med.

[22] Bischoff LL, Otto AK, Hold C, Wollesen B (2019). The effect of physical activity interventions on occupational stress for health personnel: a systematic review. Int J Nurs Stud.

[23] Ciezar-Andersen SD, Hayden KA, King-Shier KM (2021). A systematic review of yoga interventions for helping health professionals and students. Complement Ther Med.

[24] La Torre G, Raffone A, Peruzzo M (2020). Yoga and mindfulness as a tool for influencing affectivity, anxiety, mental health, and stress among healthcare workers: results of a single-arm clinical trial. J Clin Med.

[25] Shaukat N, Ali DM, Razzak J (2020). Physical and mental health impacts of COVID-19 on healthcare workers: a scoping review. Int J Emerg Med.

[26] Sharma K, Anand A, Kumar R (2020). The role of yoga in working from home during the COVID-19 global lockdown. Work.

[27] Raj Lakshmi RK, Oinam E (2021). Impact of yoga on the work-life balance of working women during COVID-19 pandemic. Front Psychol.

[28] Sarkar S, Sa B, Singh K (2021). Psychophysiological effects of yoga on stress management among medical and allied health professional students during COVID-19 pandemic: a narrative review. Adv Hum Biol.

[29] Mandal S, Misra P, Sharma G (2021). Effect of structured yoga program on stress and professional quality of life among nursing staff in a tertiary care hospital of Delhi-a small scale phase-II trial. J Evid Based Integr Med.

[30] Muralidharan S, Lakshmi S, Nath S (2022). Effect of yoga on physical and psychological wellbeing in healthcare workers during the covid.

[31] Dos Santos GM, Verlengia R, Ribeiro AG, Corrêa CA, Ciuldim M, Crisp AH (2022). Yoga and mental health among Brazilian practitioners during COVID-19: An internet-based cross-sectional survey. Sports Med Health Sci.

[32] Stephens I (2017). Medical yoga therapy. Children (Basel).

[33] Lauche R, Langhorst J, Lee MS, Dobos G, Cramer H (2016). A systematic review and meta-analysis on the effects of yoga on weight-related outcomes. Prev Med.

[34] Misra P, Sharma G, Tandon N (2021). Effect of community-based structured yoga program on HbA1c level among type 2 diabetes mellitus patients: an interventional study. Int J Yoga.

[35] Ghazvineh D, Daneshvar M, Basirat V, Daneshzad E (2022). The effect of yoga on the lipid profile: a systematic review and meta-analysis of randomized clinical trials. Front Nutr.

